# Plasma profile of microRNA after supplementation with high doses of vitamin D_3_ for 12 months

**DOI:** 10.1186/1756-0500-5-245

**Published:** 2012-05-17

**Authors:** Rolf Jorde, Johan Svartberg, Ragnar Martin Joakimsen, Dag H Coucheron

**Affiliations:** 1 Tromsø Endocrine Research Group, Department of Clinical Medicine, University of Tromsø, Tromsø, Norway and Division of Internal Medicine, University Hospital of North Norway, 9038, Tromsø, Norway; 2 RNA and Transcriptomics Group, Department of Medical Biology, University of Tromsø, Tromsø, Norway

**Keywords:** microRNA, Randomized clinical trial, Vitamin D

## Abstract

**Background:**

Recently a large number of short non-coding-RNAs (microRNAs, (miRNA)) have been identified. These miRNAs act as post-transcriptional regulators where they generally have an inhibitory function. miRNAs are present in all human cells, and they are also detected in serum or plasma. The miRNAs have a broad range of actions, and their biogenesis must therefore be under tight control. One putative regulator of miRNA biogenesis or miRNA level could be vitamin D, an ancient hormone with effects on cell growth and differentiation, apoptosis and the immune system. In our study miRNA were reversed transcribed in total RNA isolated from plasma and analyzed by quantitative real-time PCR (qPCR) using the miRCURY LNA Universal RT microRNA PCR system (Exiqon). In 10 pilot subjects 136 miRNAs were detected in one or more plasma samples drawn at baseline and after 12 months of vitamin D supplementation. The twelve miRNAs that showed the greatest change in expression in these pilots were further analyzed by RT-qPCR of RNA from baseline and 12 months plasma samples in 40 subjects given high dose vitamin D_3_ (20.000 – 40.000 IU per week) and 37 subjects given placebo.

**Results:**

At baseline there was a significant and positive correlation between serum 25-hydroxyvitamin D and miR-532-3p expression (r = 0.24, P = 0.04). The change in expression of miR-221 from baseline to 12 months (ddCp value) was also significantly different between the vitamin D and placebo group (P =0.04), mainly due to a change in the placebo group.

**Conclusions:**

We have not been able to demonstrate a consistent effect of vitamin D supplementation on the expression profile of miRNA in plasma. However, further studies are needed as this approach might potentially throw light on unknown aspects of vitamin D physiology.

## Background

It has recently been found that a large part of the human genome is transcribed into non-coding RNAs with major functions both in normal physiology and in pathological processes [1,2]. One group of these non-coding-RNAs is the small regulatory RNAs of which microRNAs (miRNAs) of ~22 nucleotides (nts) in size have been the most studied [3]. So far more than1500 miRNAs have been annotated in humans in miRBase v.18 and the number is still increasing [4].

The function of miRNAs appears to be in gene regulation where they act as post-transcriptional regulators. For that purpose, a miRNA is complementary to a part of one or more messenger RNAs (mRNAs). Perfect or near perfect base pairing with the target RNA promotes cleavage and destruction of the mRNA, whereas miRNAs that are only partially complementary to the target may inhibit protein translation of mRNA and also cause the mRNAs to be degraded sooner [5]. The mRNA is thus “silenced” and the protein coded for not produced. miRNAs therefore generally have an “inhibitory” function [6].

miRNAs are present in all human cells, are reported to target 30 - > 60% of all protein coding genes [7,8] and the expression profile differs from tissue to tissue [9]. miRNAs appear to have all sorts of functions in physiology, from cell differentiation, proliferation, and apoptosis [10] to the regulation of the endocrine system and metabolism [11,12]. Over and under-expression of miRNA appear to occur and have been linked to various human diseases, like CNS disorders [13], vascular diseases [14], viral diseases [15], diabetes [4], and in particular to cancers [16]. In general, these associations are based on studies in tissue samples. However, such tissue samples are not easily obtained, and it was therefore a major step forward when it was found that miRNAs are also detected, and remarkably stable, in serum or plasma [17]. Furthermore, it was found that in certain diseases like cancer and diabetes miRNAs in the serum may serve as potential biomarkers [18-20], and the specific serum miRNA expression profile, or a single or a couple of miRNA(s), may constitute a fingerprint of a physiological condition.

Given the broad action of miRNAs, regulation of miRNA biogenesis must be under tight control. However, so far this has not been extensively studied, but control mechanisms appear to operate both at transcriptional and post-transcriptional levels [21]. One putative regulator of miRNA biogenesis could be vitamin D, and a change in vitamin D concentration could alter the level and number of miRNAs. Vitamin D is a hormone found in all species with a calcified skeleton. It is essential for life in higher animals, and is the primary biological regulator of calcium homeostasis [22]. The main source of vitamin D is solar UV-radiation. Natural dietary sources of vitamin D are few, but it is found in fat fish like salmon and herring. Both dietary and solar vitamin D undergo a hydroxylation in the liver to 25-hydroxyvitamin D (25(OH)D) which is the biochemical marker that best reflects the body’s vitamin D status. It serves as a substrate for 1-α-hydroxylase in the kidneys, which under tight regulation forms the active form of the vitamin 1,25-dihydroxyvitamin D (1,25(OH)_2_ D). 1,25(OH)_2_ D affects the biological activity not only in the skeleton, but in a number of tissues [23]. It regulates key components governing cell growth, differentiation, apoptosis and immune system maturation [22], and low serum levels of 25(OH)D have been associated with mortality rate [24], cancer [25], cardiovascular diseases [26], immunological diseases [27], and diabetes [28]. 1,25(OH)_2_ D binds to a nuclear vitamin D receptor (VDR) and also to a cell membrane receptor [22]. In a review from 2005 summarizing non-calcemic actions of vitamin D, 12 genes were identified to be up-regulated and 11 genes down-regulated by VDR ligands [29]. As expected, most of these genes were related to bone remodelling, cell differentiation, metabolism and inflammation. Non-genomic effects of 1,25(OH)_2_ D, mediated by its cell membrane receptor, include opening of ion channels, which leads to activation of second messengers like PKC, PKA, and MPA kinases. These second messenger may in turn also have effects on gene expression [30].

Considering the ancient origin of vitamin D, the receptor being present in virtually all human tissue, and the similarities between known effects of vitamin D and conditions with altered miRNA profile, it is tempting to speculate that vitamin D may also function as a regulator of miRNA expression, which in turn may be reflected by altered levels of miRNA in plasma. Since we have recently performed intervention studies with high doses of vitamin D, we had the opportunity to test that hypothesis.

## Results

### Profiling of miRNAs in plasma from subjects given vitamin D_3_

To profile miRNA in plasma from subjects before (baseline) and 12 months after supplementation with 40.000 IU vitamin D_3_ per week, two pilot studies were carried out. Their baseline and 12 months characteristics are shown in Table 1. In the first pilot 136 miRNAs were detected by RT-qPCR in one or more of the samples, and 11 miRNAs showed a significant difference in mean expression value between baseline and 12 months (P < 0.05, student’s *t*-test) (Table 2). In the second pilot 113 miRNAs were detected in one or more of the samples. Eighteen of these miRNAs showed a significant difference in mean expression value between baseline and after 12 months (P < 0.05) (Table 3).

**Table 1:** **Characteristics at baseline and after 12 months of 40.000 IU vitamin D**_**3**_**per week in the two pilot groups**

	**Pilot group 1______________________________**		**Pilot group 2______________________________**	
	**Baseline**	**12 months**	**Baseline**	**12 months**
N	5	5	5	5
Age (years)	43.6 ± 9.0		55.6 ± 8.1	
Smokers (%)	0	0	0	0
BMI (kg/m^2^)	32.0 ± 1.2	31.8 ± 1.5	31.1 ± 1.3	31.0 ± 1.3
Systolic blood pressure (mmHg)	128.4 ± 20.0	128.0 ± 16.7	134.2 ± 21.9	129.0 ± 10.8
Diastolic blood pressure (mmHg)	78.0 ± 9.0	77.8 ± 13.1	81.2 ± 12.9	75.8 ± 9.0
HbA_1c_ (%)	5.56 ± 0.27	5.84 ± 0.32	5.74 ± 0.32	5.82 ± 0.33
Serum PTH (pmol/L)	5.54 ± 2.05	5.38 ± 1.43	5.36 ± 1.28	4.08 ± 0.95
Serum 25(OH)D (nmol/L)	41.5 ± 6.3	107.7 ± 7.3	57.8 ± 6.7	127.8 ± 16.0

Values are mean ± SD.

**Table 2:** **Plasma miRNAs with significant change in expression after 1 year of 40.000 IU vitamin D**_**3**_**per week in the first five pilot subjects**

**miR**	Fold change from baseline*	P value 12 month vs baseline**
let-7f	1.35	0.04
miR-106b	0.68	0.04
miR-133b	1.54	0.03
miR-19a	0.81	0.01
miR-22	0.79	0.02
miR-26a	1.40	0.01
miR-28-5p	1.79	0.02
miR338-3p	1.91	0.03
miR-424	0.89	0.05
miR-548b-3p	0.41	0.01
miR-660	0.79	0.03

*A fold change >1 indicates up regulation after 12 months, whereas a value <1 indicates down-regulation after 12 months; **Paired *t*-test.

**Table 3:** **Plasma miRNAs with significant change in expression after 1 year of 40.000 IU vitamin D**_**3**_**per week in the last five pilot subjects**

**miR**	Fold change from baseline*	P value 12 month vs baseline**
let-7a	1.43	0.01
Let-7d	1.57	0.02
let-7f	1.59	0.02
miR-146a	1.46	0.04
miR-151-3p	1.28	0.02
miR-151-5p	1.39	0.01
miR-15b	1.37	0.04
miR-191	1.59	0.02
miR-221	1.63	0.01
miR-26a	1.96	0.02
miR-324-5p	0.59	0.03
miR-331-3p	1.45	0.02
miR-339-5p	1.60	0.03
miR-374b	1.43	0.03
miR-532-3p	0.47	0.01
miR-543	1.79	0.03
miR-766	2.08	0.01
miR-99b	1.82	0.01

*A fold change >1 indicates up regulation after 12 months, whereas a value <1 indicates down-regulation after 12 months; **Paired *t*-test.

### Analysis of twelve selected plasma miRNAs, serum 25(OH)D and serum PTH from subjects given vitamin D_3_

Two of the miRNAs were significantly different in both pilots (miR-26a and let-7f). These two miRNAs and those where the difference between mean values at baseline and after 12 months had a P value ≤ 0.02 (4 in the first pilot and 6 in the second) were selected for the main study.

These 12 miRNAs were analyzed by RT-qPCR in plasma from 40 subjects given vitamin D_3_ (19 given 20.000 and 21 40.000 IU per week) and in 37 subjects given placebo. The subjects given 20.000 IU and 40.000 IU vitamin D_3_ per week are presented as one vitamin D group. The baseline and 12 months characteristics of the vitamin D and placebo groups are given in Table 4. As expected, the group given vitamin D had a substantial increase in serum 25(OH)D and a significant decrease in serum PTH levels, which was not seen in the placebo group.

**Table 4:** Characteristics at baseline and after 12 months in the vitamin D and placebo group

	Vitamin D group*______________________________		**Placebo group______________________________**	
	**Baseline**	**12 months**	**Baseline**	**12 months**
N	40	40	37	37
Age (years)	48.9 ± 10.0		51.0 ± 10.1	
Smokers (%)	20.0		18.9	
BMI (kg/m^2^)	34.4 ± 4.2	34.5 ± 4.5	34.3 ± 3.1	34.5 ± 3.5
Systolic blood pressure (mmHg)	130.7 ± 13.6	136.2 ± 16.9	130.9 ± 16.1	131.6 ± 16.6
Diastolic blood pressure (mmHg)	81.5 ± 10.8	84.1 ± 10.5	79.4 ± 10.5	80.3 ± 8.9
HbA_1c_ (%)	5.62 ± 0.40	5.74 ± 0.40	5.74 ± 0.42	5.80 ± 0.46
Serum PTH (pmol/L)	5.63 ± 1.60	4.64 ± 1.55^a^	5.79 ± 1.81	5.66 ± 2.06
Serum 25(OH)D (nmol/L)	50.2 ± 14.2	101.7 ± 17.8^a^	53.0 ± 19.1	49.6 ± 16.0

*19 subjects given 20.000 IU and 21 subjects given 40.000 IU vitamin D_3_ per week. ^a^ P <0.01 versus baseline in the vitamin D group and versus 12 months values in the placebo group; t-tests. Values are mean ± SD

Two of the 12 selected miRNAs (miR-19a and miR-548b-3p) were detected in only a few of the samples due to low expression and not included in the following presentation. At baseline there were no significant differences between the two groups regarding the expression of the miRNAs. In the placebo group there was a significant (P = 0.03) decrease in median miR-221 expression from 0.02 at baseline to – 0.28 after 12 months, and the change in miR-221 expression from baseline to 12 months (ddCp value) was also significantly different between the two groups (P = 0.04) (Table 5). There were no other significant changes for any of the other miRNAs in any of the two groups. At baseline there was a significant and positive correlation between serum 25(OH)D and miR-532-3p expression (r = 0.24, P = 0.04). There were no correlations between serum 25(OH)D and miRNAs regarding changes from baseline to 12 months.

**Table 5:** miRNA expression. Average expression values at baseline, after 12 months and average expression change from baseline (delta values) in the vitamin D and placebo groups

	Vitamin D group*___________________________________________			**Placebo group___________________________________________**		
	**Baseline average expression (dCp)**	**12 months average expression (dCp)**	^**a**^**Average difference in expression (ddCp)**	**Baseline average expression (dCp)**	**12 months average expression (dCp)**	^**a**^**Average difference in expression (ddCp)**
let-7f	0.14 (−0.06, 0.39)	0.29 (−0.10, 0.45)	0.11 (−0.38, 0.37)	0.10 (−0.05, 0.30)	0.11 (−0.12, 0.29)	−0.12 (−0.34, 0.22)
let-7a	1.09 (0.86, 1.18)	1.06 (0.88, 1.27)	0.07 (−0.15, 0.26)	0.94 (0.82, 1.17)	1.02 (0.81, 1.19)	0.06 (−0.23, 0.23)
miR-151-5p	−0.88 (−1.12, -0.68)	−0.90 (−1.08, -0.66)	0.04 (−0.26, 0.24)	−0.92 (−1.24, -0.76)	−1.14 (−1.35, -0.78)	−0.12 (−0.47, 0.17)
miR-22	−0.81 (−1.13, -0.57)	−0.91 (−1.17, -0.56)	−0.09 (−0.38, 0.29)	−0.72 (−1.12, -0.49)	−0.79 (−0.97, -0.48)	−0.04 (−0.19, 0.25)
miR-221	0.03 (−0.32, 0.29)	0.09 (−0.21, 0.27)	0.13 (−0.21, 0.29)	0.02 (−0.27, 0.26)	−0.28 (−0.58, 0.10)	−0.32 (−0.53, 0.10)
miR-26a	2.07 (1.90, 2.24)	2.09 (1.81, 2.25)	−0.02 (−0.29, 0.26)	1.94 (1.70, 2.11)	1.83 (1.53, 2.15)	−0.05 (−0.39, 0.25)
miR-28-5p	−4.10 (−4.51, -3.66)	−4.11 (−4.48, -3.71)	0.09 (−0.60, 0.53)	−4.14 (−4.63, -3.93)	−4.30 (−4.55, -3.95)	−0.14 (−0.76, 0.32)
miR-532-3p	−4.80 (−5.05, -4.37)	−4.68 (−4.84, -4.25)	0.06 (−0.46, 0.63)	−4.66 (−5,21, 4.24)	−4.63 (−5.03, -4.27)	0.19 (−0.45, 0.42)
miR-766	−3.53 (−3.97, -3.30)	−3.59 (−4.01, -3.32)	−0.11 (−0.39, 0.39)	−3.68 (−4.00, -3.41)	−3.72 (−4.14, -3.44)	−0.10 (−0.39, 0.18)
miR-99b	−3.42 (−3.61, -3.12)	−3.34 (−3.73, -3.04)	0.03 (−0.42, 0.27)	−3.43 (−3.75, -3.14)	−3.42 (−3.91, -3.23)	0.04 (−0.33, 0.20)

*19 subjects given 20.000 IU and 21 subjects given 40.000 IU vitamin D_3_ per week. ^a^ Average difference in expression values (average expression at 12 months minus average expression at baseline); ^b^ P = 0.03 vs baseline (Wilcoxon signed rank test); ^c^ P = 0.04 vs placebo group (Mann–Whitney U- test). Values are median (25, 75 percentile)

## Discussion

To our knowledge this is the first report on potential effect of vitamin D on miRNA profile in plasma. At baseline we found a significant but weak correlation between miR-532-3p expression and serum 25(OH)D that was most probably due to chance. Furthermore, there was a difference between the placebo group and the vitamin D group regarding change in miR-221 expression from baseline to 12 months, but this was due to a reduction in miR-221 in the placebo group whereas that miRNA was unchanged in the vitamin D group. Accordingly, we were not able to demonstrate any consistent and truly significant effect of vitamin D on the miRNA plasma profile.

We only analyzed 12 miRNA in the main study. Ideally, we would have preferred to do a complete miRNA profiling by RT-qPCR in all the 77 subjects, but for financial reasons that was not possible. An additional factor was that inclusion of 100–150 miRNAs would have made it very difficult to evaluate whether an observed change was truly significant or simply due to chance because of a large number of miRNA analyzed. Instead we did two pilot studies and included the 12 most promising miRNAs. Two of these miRNAs had shown a significant expression change from baseline to 12 months in both pilot studies and an additional ten miRNAs had shown a significant change with P ≤ 0.02 in one of the pilot studies. We therefore anticipated that we could reproduce a significant effect of vitamin D for at least a few of these miRNAs. Since information is available on which gene(s) the miRNAs are targeting this could potentially disclose new aspects of vitamin D’s role in biology.

However, we could not reproduce the findings from the pilot studies, nor did we find any new consistent correlations. For this there could be a number of reasons. First of all, we might have analyzed the “wrong” miRNAs. Although there are no previous reports on effect of vitamin D supplementation on plasma or serum miRNA profile, there are reports on effects of vitamin D on cellular miRNA expression. Thus, in a prostate cancer cell line (LNcaP) 15 miRNAs were either up- or down-regulated by 1,25(OH)_2_D [31]. Among these 15 miRNAs, only miR-22, which was up-regulated, was included in our analysis. Furthermore, in a study on early pregnancy where RNA had been extracted and purified from whole blood, women with low versus high serum 25(OH)D levels had different expression of 11 miRNAs [32]. However, none of these eleven miRNAs were among those analyzed by us. And finally, in a study on breast epithelial cells, 25(OH)D was found to protect against cellular stress, which might be mediated by altered expression of five miRNAs [33]. But again, none of these miRNAs were analysed in our study.

Secondly, the amount of miRNA in plasma is often low as demonstrated by the number of cycles needed before detection in the RT-qPCR assay. We observed variation in the expression profiles of miRNAs between first and second pilot study (e.g. between baselines) and in the expression of individual miRNA both in the baseline and 12 months samples. Normal biological variation of miRNA levels in plasma together with variation introduced during sample treatments (from blood sampling to RT-qPCR) may affect the observed expression of miRNAs [34]. Also in the initial report on miRNA in serum, most of the miRNAs were detected in both serum and blood cells. However, only a small number of miRNAs were uniquely present in either serum or blood cells, indicating that under normal conditions most serum miRNAs are derived from circulating blood cells [17]. Accordingly, in our subjects, who were healthy apart from their overweight, an effect of vitamin D might not be easily detected. Furthermore, most of our subjects had normal serum 25(OH)D levels, and it is plausible that an effect of vitamin D is most evident in vitamin D deficiency. It should also be considered that an effect of vitamin D could have been present at an earlier time point than 12 months, and as the effect of vitamin D may be U-shaped [24], lower doses of vitamin D_3_ might also have been more effective.

On the other hand, our study also has some strength. We included a large group of subjects, and the vitamin D doses given were high and the resulting serum 25(OH)D levels were in the upper physiological range [22]. In addition, the miRCURY LNA RT-qPCR system used for detection of known plasma miRNAs has been found to be more sensitive and reproducible than microarray systems and show even more sensitivity and linearity than TaqMan methods at low concentration of plasma miRNA [35,36].

## Conclusion

In conclusion, we have not been able to demonstrate an effect of vitamin D supplementation on the miRNA plasma profile expression. However, further studies are needed as this approach might potentially throw light on unknown aspects of vitamin D physiology.

## Methods

### Selection of subjects, study design and blood sampling

Plasma samples used in the pilot and main study were all randomly selected from a previous study where we evaluated the effect of high doses of vitamin D supplementation (20.000 IU or 40.000 IU vitamin D_3_ per week) versus placebo for one year on weight reduction in obese subjects [37]. Only males were included in the present study, all subjects were generally in good health, and none had diabetes or any other serious concomitant diseases. Fasting blood samples were drawn at baseline and after 12 months at the end of the study. Blood for plasma preparation was collected using a 19-gauge needle into vacutainers (Becton Dickinson, Meylan Cedex, France) containing 0.129 M sodium citrate (1 volume anticoagulant and 9 volumes whole blood) as anticoagulant, centrifuged at 2000 g for 15 minutes at 22°C, transferred into sterile cryovials in aliquots of 1 ml and stored at −70°C until further analysis.

**Figure 1:**
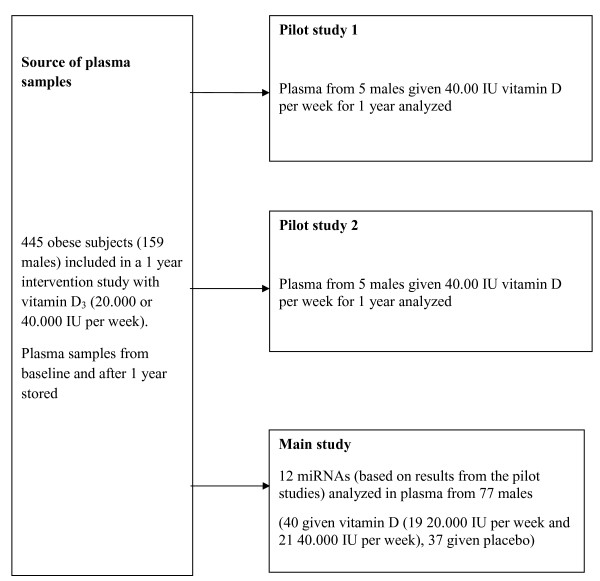
Box chart showing the experimental design.

To identify miRNAs potentially affected by vitamin D supplementation, two pilot studies each including plasma samples from five subjects given 40.000 IU vitamin D_3_ were performed. The most promising miRNAs were then selected and baseline and 12 months plasma samples analysed in 40 subjects given vitamin D_3_ (19 given 20.000 IU and 21 40.000 IU per week) and 37 subjects given placebo. A box chart showing the experimental design is presented in Figure 1.

### RNA isolation, reverse transcription and quantitative real-time PCR

All the total RNA isolations and real-time qPCR experiments were performed by “Exiqon Services”, Vedbaek, Denmark. Total RNA was isolated from 200 μl plasma samples using the QIAGEN miRNeasy Mini Kit Protocol (Quiagen) slightly modified by Exiqon (http://www.exiqon.com/ls/Documents/Scientific/serum-plasma-RNA-isolation.pdf) with elution in 50 μl DNase/RNase-free water. Two μl of total RNA was reverse transcribed in 10 μl reactions using the miRCURY LNA™ Universal RT miRNA PCR, Polyadenylation and cDNA synthesis kit (Exiqon). Four ul of cDNA diluted 50 x (equivalent to 0.064 μl original plasma sample), was assayed in 10 ul PCR reactions according to the protocol for miRCURY LNA™ Universal RT miRNA PCR. DNA and RNA spike-ins were included in the qPCR and RT step, respectively. Negative controls, without enzymes, from the reverse transcription reaction was treated and profiled like the samples. All amplifications were performed in a LightCycler® 480 Real-Time PCR System (Roche) in 384 well plates.

In the two pilots studies plasma samples from baseline and after 12 months were analyzed for presence of miRNA. Each cDNA sample was assayed once by qPCR on the microRNA Ready-to-Use PCR, Human panel I and panel II containing 730 miRNAs (first pilot) and 742 mRNAs (second pilot).

In the main study plasma from the 77 subjects were analysed for expression of 12 miRNAs (let-7a, let-7f, miR-19a, miR-22, miR26a, miR28-5p, miR-99b, miR151-5p, miR-221, miR-532-3p, miR-548-3p, miR-766). Each plasma sample was processed in singlicates and each cDNA (miRNA) sample was assayed once by qPCR.

### Data pre-processing

Determination of Cp values and the generation of amplification- and melting curves were performed by use of the The LightCycler® 480 software. Amplification efficiency was determined by the LinRegPCR (ver 11.5) software. All assays were inspected for distinct melting curves and T_m_ was checked to be within known specifications for the assay. Only assays detected with 5Cp’s less than the negative control and with Cp < 37 were included in the data analysis. Data that did not pass these criteria were omitted from any further analysis. The average amplification efficiency was used to correct the Raw Cp values. For the pilot studies NormFinder found the geomean of miRNAs detected in all samples to be the best normalizer. All data was normalized to the geomean using the formula Normalized Cp = Geomean (n = 10) – assay Cp. For the main study the data was normalized based on the geomean Cp of the reference miRNAs miR-30a, miR-103, miR-191, miR-320a and miR-423-5p, on a well-to-well basis. The efficiency corrected Cps of the five reference miRNAs in each plasma sample are given in the Additional file 1: Table S1.

### Other analyses

Height and weight, blood pressure, serum parathyroid hormone (PTH) and 25(OH)D were measured/analyzed as previously described [37].

### Statistical analyses

Normal distribution was evaluated with kurtosis and skewness and visual inspection of histograms. The variables age, BMI, blood pressure, serum PTH and 25(OH)D were normally distributed whereas several of the miRNAs were not. As these miRNAs also included negative numbers, logarithmic transformation was not possible. Therefore, non-parametric statistics were used for the miRNAs. Comparisons between the vitamin D group and the placebo group were done with student’s *t*-test or Mann–Whitney U- test, and within each group with paired student’s *t*-test or Wilcoxon signed rank test. Correlations were evaluated with Spearman’s rho. When comparing the effects of vitamin D versus placebo after 12 months, the ddCp values were used (value at 12 months minus value at baseline). The data are presented as mean ± SD or median (25, 75 percentile). All tests were done two-tailed and P < 0.05 was considered statistically significant. The P-values are presented without correction for multiple testing.

### Ethics

The study was approved by the Regional Committee for Medical and Health Research Ethics, North Norway (REK NORD). Only participants with valid written consent were included.

## Abbreviations

BMI, Body mass index; MRNAs, Messenger RNAs; miRNA, microRNA; PTH, Parathyroid hormone; VDR, Vitamin D receptor; 1,25(OH)2 D, 1,25-dihydroxyvitamin D; 25(OH)D, 25-hydroxyvitamin D.

## Competing interests

The authors declare that they have no competing interests.

## Authors’ contributions

All authors made substantial contributions to conception and design, analysis and interpretation of data. RJ made the initial draft of the manuscript. JS, RMJ and DC revised it critically for important intellectual content. All authors have read and given final approval of the version to be published.

## Supplementary Material

Additional file 1 **Table S1. **A sheet containing the efficiency corrected Cps of the reference miRNAs. Click here for file
